# Successful Nonsurgical Management of *Clostridium perfringens* Sepsis With Massive Intravascular Hemolysis and Liver Abscess: A Case Report

**DOI:** 10.1155/crdi/1328614

**Published:** 2025-09-23

**Authors:** Diogo Costa Oliveira, Inês Mendonça, Carolina Vaz-Pinto, Cristina Marques, Alexandra Babo, Vasco Elói

**Affiliations:** ^1^Serviço de Medicina Intensiva, Unidade Local Saúde Trás-os-Montes e Alto Douro, Vila Real, Portugal; ^2^Serviço de Medicina Intensiva, Unidade Local Saúde São João, Porto, Portugal; ^3^Serviço de Patologia Clínica, Unidade Local Saúde São João, Porto, Portugal; ^4^Serviço de Cirurgia Geral, Unidade Local Saúde São João, Porto, Portugal

**Keywords:** case report, *Clostridium perfringens*, fulminant hemolysis, liver abscess, percutaneous drainage

## Abstract

**Background:** *Clostridium perfringens* is an anaerobic, Gram-positive, spore-forming bacterium, commonly associated with gas gangrene and clostridial myonecrosis. Although bacteremia is rare, it carries a high mortality rate, particularly when complicated by massive intravascular hemolysis.

**Case Presentation:** We report the case of a woman with poorly controlled diabetes mellitus who presented with fever, abdominal pain, systemic inflammatory response syndrome, respiratory insufficiency, and hemolytic anemia. Imaging studies revealed a hepatic abscess with gas formation, and blood cultures confirmed *C. perfringens* sepsis. Due to the patient's critical status, surgical intervention was deferred, and a multidisciplinary team initiated treatment involving intensive care, hematology, microbiology, immunohemotherapy, and surgery.

**Management and Outcome:** Treatment included high-dose beta-lactam antibiotics combined with clindamycin, chosen for its antitoxin properties, alongside percutaneous drainage of the liver abscess and comprehensive intensive care support, including renal replacement therapy. The patient demonstrated progressive clinical improvement, with resolution of hyperlactatemia, successful weaning from vasopressors, and extubation. She completed a 28-day course of antibiotics and was discharged after a 75-day hospital stay.

**Conclusion:** This case highlights the vital importance of early clinical suspicion, prompt diagnosis, and coordinated multidisciplinary management in *C. perfringens* sepsis. In the absence of well-established, evidence-based treatment protocols for this fulminant infection, our report illustrates a successful nonsurgical approach combining timely antimicrobial therapy, percutaneous source control through interventional radiology, and intensive organ support. Further research is essential to better define optimal management strategies and improve outcomes in this life-threatening condition.

## 1. Introduction

*Clostridium perfringens* is an obligate, anaerobic, Gram-positive, spore-forming, and toxin-producing bacterium commonly found in the gastrointestinal and genital human tracts and the environment. Infections can occur when a wound is contaminated under conditions favorable for bacterial proliferation, such as hypoxia and acidity, leading to gas gangrene or clostridial myonecrosis [1].

Bloodstream infections caused by this organism are uncommon, predominantly affecting individuals with cancer or immunocompromised states [2] and are associated with an exceedingly high mortality rate, making it one of the most severe forms of bacteremia [1]. It is even more devastating when the bacteremia is associated with massive intravascular hemolysis, which occurs in 7%–15% of the cases [3].

Mortality in *C. perfringens* sepsis exceeds 70%, and optimal treatment strategies remain poorly defined due to the rarity of favorable outcomes [4]. In this case report, we describe a rare instance of spontaneous, nontraumatic hepatic gas gangrene caused by *C. perfringens*, detailing our diagnostic workup and multidisciplinary management. Our approach included early initiation of antimicrobial therapy with antitoxin activity, an attempted percutaneous source control strategy, and intensive organ support, ultimately resulting in patient survival.

## 2. Case Presentation

A woman in her 60s with a medical history of hypertension, dyslipidemia, and poorly controlled type 2 diabetes mellitus (glycated hemoglobin [HbA1C] of 7.7% 6 months prior to presentation) on four oral hypoglycemic agents presented at a district hospital emergency department with a one-day history of constant epigastric and right hypochondrial pain with dorsal irradiation. She reported no association of the pain with meals and had no similar prior episodes. Apart from the pain, she had no additional complaints, denying fever, nausea, vomiting, or diarrhea. Initial laboratory tests were only notable for elevated lipase levels (refer to Table 1), leading to a presumed diagnosis of acute pancreatitis; fluid therapy and pain management medications were initiated, and the patient was admitted for further observation. Abdominal ultrasound revealed a liver of normal morphology and dimensions, a gallbladder with a markedly thickened and irregular wall (6 mm), nondistended and with apparent cholelithiasis, and intrahepatic and extrahepatic bile ducts of standard caliber.

By the third day of admission, the patient's pain persisted, and she developed fever, moderate hypoxic respiratory insufficiency, and metabolic acidosis accompanied by hyperlactatemia (refer to Table 1). Additionally, a significant drop in hemoglobin levels was noted (from 15.1 at admission to 4.8 g/dL); further analysis was impaired as multiple blood specimens were found to be hemolysed. One analyzed sample showed elevated red cell distribution width (RDW), a high percentage of reticulocytes, and newly onset hyperbilirubinemia, predominantly unconjugated, raising suspicions of hemolytic anemia.

**Figure 1:**
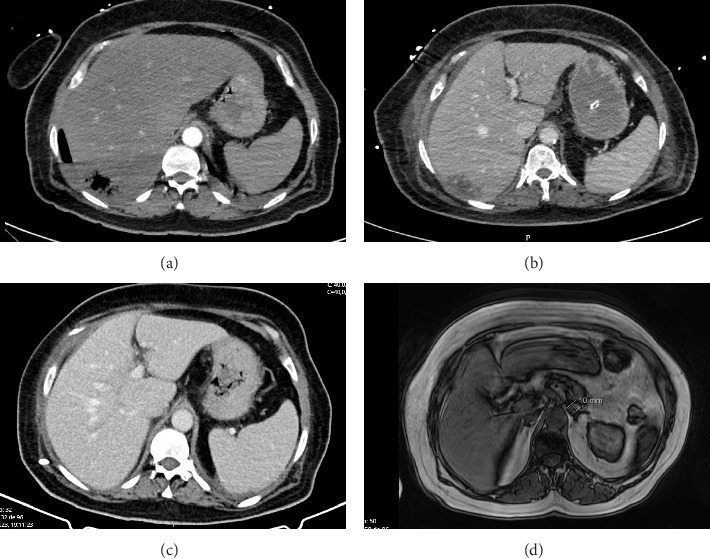
Imaging evolution of the hepatic abscess. (a) (Admission): abdominal computed tomography (CT) showing a VII segment hepatic abscess; (b) (3 days after admission): CT showing the hepatic abscess 54 × 30 mm with a pigtail drain; increment of the liquid component of the abscess; (c) (one-month after admission): CT showing decrease of the VII segment hepatic abscess (37 × 17 mm), pigtail drain inside; and (d) (9-month follow-up after discharge): magnetic resonance imaging T1 showing hepatomegaly with signs of steatosis; complete resolution of the abscess previously viewed in segment VII.

The peripheral blood smear showed frequent spherocytes, rare erythrocyte fragments, polychromasia, erythrocytes with basophilic stippling, and moderate erythrocyte agglutination. Considering the possibility of immune-mediated hemolytic anemia, an IV bolus of methylprednisolone (equivalent to 1 mg/kg of prednisolone) was administered. However, both direct and indirect Coombs tests, which were later performed, returned negative results. Despite an unremarkable abdominal ultrasound conducted the previous day, a subsequent abdominal CT scan revealed a 51 × 26 mm hepatic abscess with gas formation in the VIII segment (see Figure 1). The patient's clinical status rapidly deteriorated with worsening mental status, respiratory distress, and oliguria, necessitating noninvasive ventilation and the need for vasopressor support. At this point, blood cultures were collected, and the patient was started on piperacillin–tazobactam and transferred to a tertiary hospital for further management in the intensive care unit (ICU).

**Figure 2:**
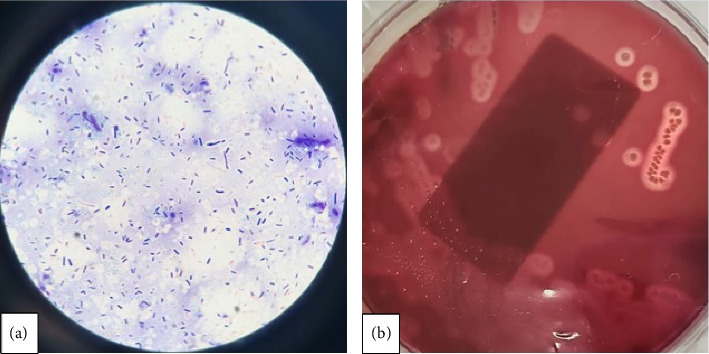
Microbiology identification of *Clostridium perfringens*. (a) Gram stain of the blood culture bottle, demonstrating Gram-positive bacilli, consistent with the posterior identification of *C. perfringens*. (b) *C. perfringens* colonies isolated from the blood culture on blood agar, inducing medium hemolysis, typically described as double zone hemolysis.

A multidisciplinary team was gathered to discuss the case, including specialists from Intensive Care Medicine, Hematology and Immunohematology, Microbiology, and General Surgery. The central diagnostic consideration was nonimmune hemolytic anemia in the context of a pyogenic liver abscess with gas. A comprehensive literature review suggested that this presentation might indicate gas gangrene secondary to *C. perfringens* infection. Although the initial Wright staining of peripheral blood did not identify neutrophils with intra- or extracellular bacilli, Gram staining of blood cultures identified Gram-positive organisms (see Figure 2). Due to the patient's instability, surgical debridement was deemed too risky. Consequently, the team opted for percutaneous drainage of the abscess. Additionally, clindamycin 900-mg IV every 8 h was incorporated into the antibiotic treatment regimen alongside piperacillin–tazobactam at 18 g/day. The patient's condition deteriorated further due to worsening respiratory distress and neurological decline, necessitating intubation and the initiation of mechanical ventilation.

In the ICU, the patient required substantial vasopressor support (reaching a maximum noradrenaline dose of 0.6 mcg/kg/min) and began continuous renal replacement therapy (hemodiafiltration) within 6 hours of admission. She received a total of six units of red blood cells. Ongoing discussions with the general surgery team were held daily due to the potential need for surgical debridement. A conservative approach was chosen, supported by frequent imaging re-evaluations, as the patient showed clinical and laboratory improvements. On Day 11, the drain required repositioning, guided by an ultrasound.

The patient's clinical course in the ICU showed favorable progress: hyperlactatemia was resolved by Day 3, vasopressor support was discontinued by Day 6, and she was successfully extubated on Day 9. Despite persistent anuria throughout her ICU stay, which necessitated ongoing renal support, she was able to transition from continuous to intermittent hemodialysis on Day 15.

The patient was transferred from the ICU to the Infectious Diseases Department on Day 15 and discharged after 75 days in the hospital. She completed a 28-day course of antibiotherapy consisting of piperacillin–tazobactam infused at 18 g/day and clindamycin administered 900 mg every 8 h. An attempt to de-escalate to penicillin was made on Day 15 of antimicrobial treatment. Still, it was reversed back to piperacillin–tazobactam and clindamycin due to clinical deterioration, marked by increased prostration and elevated inflammatory markers. The pigtail catheter was removed on Day 38 of treatment. Magnetic resonance imaging on Day 56 of hospitalization revealed a reduction in the size of the liver abscess to 33 × 17 mm from the initial 37 × 17 mm observed in the earlier CT scan (see Figure 1). Renal function showed improvement, allowing the cessation of hemodialysis on Day 42. Upon discharge, the patient was transferred to a rehabilitation center for further recovery and rehabilitation.

**Figure 3:**
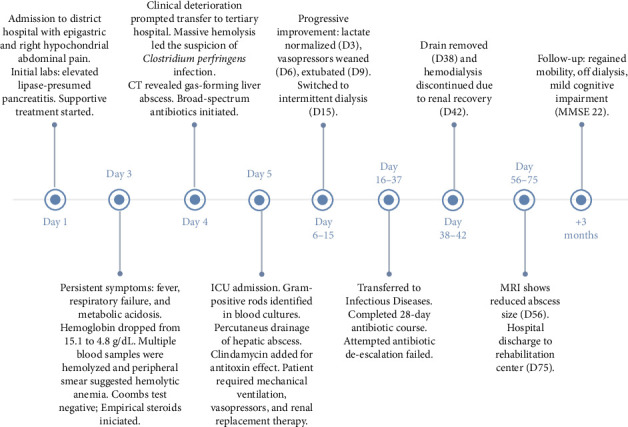
Chronological overview of the patient's clinical progression.

Three months after her discharge, the patient was seen in the outpatient clinic by the nephrology team, as well as by the infectious diseases and intensive care follow-up teams. At that time, she had returned home and made significant progress in her recovery. She had largely recovered from the acquired ICU weakness and was able to walk independently, relying only on a walking stick for support when standing. She was also no longer dependent on hemodialysis. However, she had not yet returned to work due to lingering physical limitations and mild cognitive impairment.  Figure 3 provides a chronological summary of the case.

## 3. Discussion

Sepsis caused by *C. perfringens* can lead to massive hemolysis due to the secretion of alpha- and theta-toxins [4]. This case underscores the importance of maintaining a high clinical suspicion for *C. perfringens* infection in patients with fulminant hemolysis and liver abscess, even in the absence of trauma or previous biliary interventions via endoscopy. In our patient, the only identified risk factor was poorly controlled diabetes mellitus, a known predisposing condition alongside alcohol use, abdominal surgery, lymphoma, leukemia, and malnutrition [5]. Rapid diagnosis is crucial to initiate early treatment and prevent progression to severe hemolysis and disseminated intravascular coagulation, which carries a high mortality rate in nearly all reported cases.

Regarding treatment, the available literature strongly supports prompt surgical debridement for gas gangrene, with evidence indicating significantly improved survival when performed within 24 h of admission [6]. However, in this case, we prioritized early initiation of broad-spectrum antibiotics, including high-dose clindamycin, due to its ability to inhibit toxin production. Unlike standard recommendations, we opted for a minimally invasive percutaneous approach, chosen in collaboration with the hepatobiliary surgical team and interventional radiology, given the patient's critical condition and severe hemolysis. Follow-up imaging demonstrated resolution of the gas gangrene with this strategy, avoiding the need for surgical intervention.

Toxin production plays a central role in the high mortality of gas gangrene. Historically, gas gangrene antitoxin has been used in trauma-associated cases, but its efficacy is limited, and it has been linked to severe allergic reactions [4]. As demonstrated by Hara A et al. in postmortem liver studies, *C. perfringens* produces type A toxins, which are implicated in massive intravascular hemolysis, gas gangrene, and cytokine storm [7]. Clindamycin inhibits the 50S ribosomal subunit, thereby reducing exotoxin synthesis, particularly alpha-toxin. Additionally, clindamycin has been shown to enhance microbial opsonisation and phagocytosis, even at subinhibitory concentrations [8]. Given its potential benefit, we incorporated high-dose clindamycin early in the treatment regimen. Although intravenous immune globulin (IVIG) has been suggested for similar reasons, its effectiveness remains uncertain.

The patient also received comprehensive multiorgan life support, including continuous renal replacement therapy, which likely contributed to managing septic shock and acute kidney injury while potentially aiding in toxin clearance. Although the long-term survival benefits of such interventions remain debated, emerging sorbent hemadsorption devices show promise in removing proinflammatory cytokines and endotoxins [9]. Hemophagocytic syndrome was also considered due to high levels of ferritin; however, the calculated HScore was 126 points (5%–9% probability), not supporting this diagnosis.

Following these early interventions—guided by high clinical suspicion based on the patient's history—along with intensive supportive care, the patient made a favorable recovery.

In conclusion, this case highlights the importance of early clinical suspicion and prompt initiation of antibiotic therapy—potentially with antitoxin activity—in suspected *C. perfringens* sepsis, even before definitive microbiological confirmation. It also illustrates the potential role of a less invasive, percutaneous approach in selected critically ill patients, challenging the conventional recommendation for immediate surgical debridement. Given the absence of standardized treatment protocols for this fulminant infection, further research and clinical trials are essential to establish evidence-based therapeutic strategies.

## Figures and Tables

**Table 1:** Laboratory results evolution in the first week of hospital admission.

	Day 1 district hospital admission	Day 3	Day 4 transfer to central hospital	Day 5 ICU	Day 7 ICU	Reference values
*Hemogram and coagulation laboratory study*						
Hemoglobin (g/dL)	15.1	4.8	4.6	9.8	7.6	N: 12.0–16.0
Hematocrit (%)	45.0	43.4	32.5	26.7	21.5	N: 35–47
RDW (%)	12.4	12.5	15.1	19.0	16.1	
RDW-SD (fL)	40.7	84.0	52	56.4	53.1	
Reticulocytes		19.7				
Platelets (μL)	192,000	141,000	117,000	87,000	55,000	N: > 150.000
Leucocytes (μL)	11,810	14,440	30,220	26,930	22,260	N: 4.000–11.000
Neutrophils (%)			93.6	92.3	82.3	
Leucocytes (%)			2,10	3.8	8.1	
Coombs						
Direct		NEG				
Indirect		NEG				
Haptoglobin (mg/dL)				29	11	N: 50–320
Coagulation						
aPTT (s)	20.2		50.9	45.3	38.3	N: 24.2–36.4
PT (s)	10.7		17.9	16.8	14.9	N: 10.2–13.5
Fibrinogen (mg/dL)			432	5511	321	N: 180–350
D-dimers			11.02			

*Biochemical laboratory study*						
Renal						
Creatinine (mg/dL)	0.77	1.64	2.12	3.07	1.82	N: 0.51–0.95
Urea (mg/dL)	45	99	118	176	75	N: 10–50
Hepatic						
AST (U/L)	235	810	1159	4966	3950	N: 10–31
ALT (U/L)	137	357	359	1777	1569	N: 10–31
g-GT (U/L)	289	228	101	98	69	N: 7–32
ALP (U/L)	109	87	78	85	169	N: 30–120
Total BT (mg/dL)	0.67	10.79	14.21	14.2	15.66	N: < .20
Direct BT (mg/dL)	0.36	1.34		6.89	8.58	N: < 0.40
LDH (U/L)	368	217	1270	12,091		N: 135–225
Lipase (U/L)	880	250		1270		
Cytolysis						
Myoglobin (ng/mL)			513	3159	6364	N: < 146
CK (U/L)			439	1133	8548	N: 10–149
C-reactive protein (mg/L)	2	27	295	616	456	N: < 3.0
Procalcitonin (ng/mL)					14.7	N: < 0.05

*Blood-gas analysis*						
pH	7.41	7.41	7.26	7.36	7.46	N: 7.35–7.45
HCO_3_ (mEq/L)	19.6	18.4	15.4	19.3	26.9	N: 22–26
Anion gap (mmol/L)				17.3	3.3	
Art. lactate (mmol/L)	0.9	6.9	13.9	9.01	2.25	N: < 2

*Other relevant results*						
Viral serology			HIV, HCV, HBV, CMV: negative			
Anemia study			Fe: 174 mcg/dL; ferritin: 14,013 ng/mL. Folic acid: 7.4 ng/mL. Vitamin B12: 849 pg/mL. Schistocytes: negative. No platelet clumping			
Metabolic and glycemic parameters			Total cholesterol: 244 mg/dL Triglycerides: 123 mg/dL HbA1c 12.3%			

## Data Availability

The authors have nothing to report.
